# Improving medical students recognizing surgery of glioblastoma removal/decompressive craniectomy via physical lifelike brain simulator training

**DOI:** 10.1186/s12909-024-05621-w

**Published:** 2024-06-06

**Authors:** Pin-Chuan Chen, Hsin-Chueh Chen, Wei-Hsiu Liu, Jang-Chun Lin

**Affiliations:** 1 Department of Machine Engineering, National Taiwan University of Science and Technology, Taipei, 106 Taiwan; 2 Department of Neurological Surgery, Tri-Service General Hospital and National Defense Medical Center, Taipei, 114 Taiwan; 3 Department of Surgery, School of Medicine, National Defense Medical Center, Taipei, 114 Taiwan; 4 Department of Radiation Oncology, Shuang Ho Hospital, Taipei Medical University, Taipei, 106 Taiwan; 5 Department of Radiology, School of Medicine, College of Medicine, Taipei Medical University, Taipei, 106 Taiwan

**Keywords:** Medical Education, Additive Manufacturing, Physical lifelike Brain Simulator, Glioblastoma removal, Decompressive Craniectomy

## Abstract

**Background:**

This study aims to investigate the benefits of employing a Physical Lifelike Brain (PLB) simulator for training medical students in performing craniotomy for glioblastoma removal and decompressive craniectomy.

**Methods:**

This prospective study included 30 medical clerks (fifth and sixth years in medical school) at a medical university. Before participating in the innovative lesson, all students had completed a standard gross anatomy course as part of their curriculum. The innovative lesson involved PLB Simulator training, after which participants completed the Learning Satisfaction/Confidence Perception Questionnaire and some received qualitative interviews.

**Results:**

The average score of students’ overall satisfaction with the innovative lesson was 4.71 out of a maximum of 5 (SD = 0.34). After the lesson, students’ confidence perception level improved significantly (t = 9.38, *p* < 0.001, effect size = 1.48), and the average score improved from 2,15 (SD = 1.02) to 3.59 (SD = 0.93). 60% of the students thought that the innovative lesson extremely helped them understand the knowledge of surgical neuroanatomy more, 70% believed it extremely helped them improve their skills in burr hole, and 63% thought it was extremely helpful in improving the patient complications of craniotomy with the removal of glioblastoma and decompressive craniectomy after completing the gross anatomy course.

**Conclusion:**

This innovative lesson with the PLB simulator successfully improved students’ craniotomy knowledge and skills.

**Supplementary Information:**

The online version contains supplementary material available at 10.1186/s12909-024-05621-w.

## Background

Glioblastoma is the most common brain malignancy in adults and causes severe neurologic symptoms, including headache, paralysis, seizure, or delirium [1]. The primary intervention for glioblastoma is surgical tumor resection, and adjuvant chemoradiotherapy plus maintenance temozolomide (TMZ) [2]. Managing brain tumors effectively demands neurosurgeons to have comprehensive knowledge, advanced surgical skills, and practical experience in handling complications [3, 4].

In certain stages of medical education, cadaveric dissection serves as a crucial teaching aid for medical students and junior surgeons. However, this method is constrained by high costs and limited availability [5]. To address the limitations associated with cadaver-based training, various methods have been developed, with virtual reality emerging as a significant alternative for simulation training [6]. Choudhury et al. [7] used virtual reality to integrate some surgical training, such as ventriculostomy, endoscopic nasal navigation, and glioblastoma removal. Delorme et al. [8] used virtual reality technology to design a virtual simulator of glioblastoma removal. Despite solving some cadaver-based training issues, virtual reality training still has limitations. Some trainees experience worsened motion sickness after training sessions [9]. Further, students lack tactile feedback in virtual reality training, where those trained solely in virtual reality often experience considerable stress during actual surgical procedures in the operating room [9].

Hence, accessible, cost-effective, and realistic teaching aids are essential for medical training. Advancements in 3D printing technology offer a solution by creating accurate body part simulators. These not only reduce the need for cadavers but also address virtual reality’s limitations, enhancing the realism and applicability of surgical practice tools for both research and training [10].

The aim of this study is to examine medical students’ feedback on an innovative course that utilizes a 3D-printed skull and brain tumor simulator for craniotomy and glioblastoma removal. This course is designed to foster interest and initial knowledge in neurosurgical procedures among medical students, thereby facilitating a smoother transition from the medical curriculum to their subsequent internships.

## Materials and methods

### Study design

This prospective study aims to improve medical students’ understanding in neurosurgical procedures via a novel training approach using 3D-printed simulators. Eligible medical students were those who had already completed the gross anatomy course. The simulation models, created using 3D printing and molding techniques, provided realistic representations of the brain and skull. These students engaged in hands-on sessions concentrating on decompressive craniectomy and glioblastoma removal, which were enhanced by a mix of instructor-led and independent practice sessions. The program’s effectiveness is assessed via interviews, satisfaction surveys, and confidence evaluations, offering insights into the student’s learning experiences and the training module’s overall impact.

### The construction of PLB simulator

**Fig. 1:**
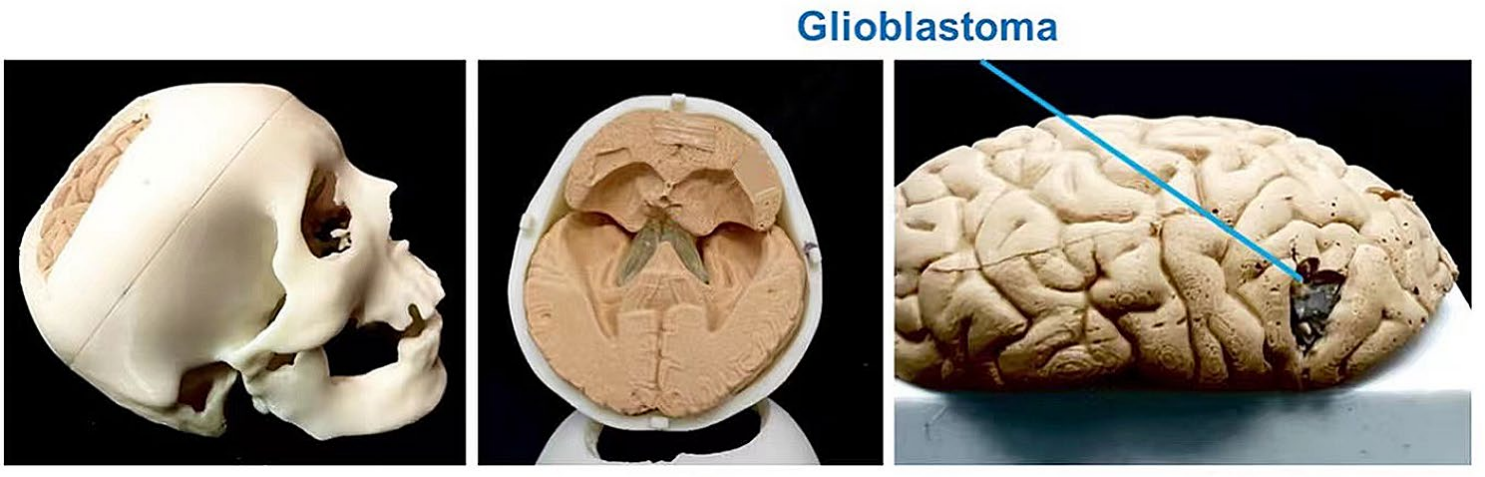
The PLB simulator. The different structures are marked in different colors: skull (white), glioblastoma (grey), and lobe (beige)

This study used low-cost and simple techniques (3D printing and casting) to develop a PLB simulator (Fig. 1). The parts of the simulator are shown as follows:

Human bone is a dense connective tissue. Bone comprises various structures, roughly divided into three parts: periosteum, bone, and bone marrow. The bone is divided into compact bone (surface layer) with a dense and hard structure and cancellous bone (inner layer) with a loose structure and is mainly composed of needle-like or sheet-like bone plates [11]. Structural analysis of compact and cancellous bone is the focus of skull fabrication. We used additive fabrication technology to simulate the layered structure of the human brain.

This step utilizes high-precision scanning technology, often employing magnetic resonance Imaging (MRI) or computerized tomography (CT) scans. We used software to analyze the structural and mechanical properties to integrate the information on the density, mass, stress, and strain of each layer of the bone structure, such as hardness and density analysis of spongy and compact bone. We combined the information obtained to analyze the relevant parameters required for 3D printing. This study used a 3D and a “stereolithographic” printer to print the skull and brain stem in acrylonitrile butadiene styrene (ABS).

The lobes are divided into four parts: frontal, parietal, temporal, and occipital lobes [11]. To reduce the difficulty of imitating the real brain, we focused on the production of the meninges and soft tissue of the brain, omitting the complex microvessels and nervous system. The process involves extensive research into the physical properties of meninges, incorporating feedback from neurosurgeons to accurately replicate the feel and elasticity of real brain tissue using silicone and other additives. Utilizing 3D printing technology, a hollow mold is crafted to form the outer shape of the brain, which is then filled with a carefully prepared mixture designed to mimic the texture and softness of both healthy brain tissue and glioblastomas. This approach ensures the bio-brain simulator not only resembles the real brain in appearance but also provides a tactile experience akin to actual surgical scenarios. As the bio-brain tissue material cures within the mold, it adopts the intricate details of the brain’s anatomy, resulting in a highly realistic simulator. We used similar methods to fabricate glioblastomas, but the jelly mix contained only glycerol and jelly candles to fabricate glioblastoma.

The cost of materials for creating the 3D models is approximately 160 USD, significantly lower than the 3300 USD required for cadaver usage. This substantial cost reduction allows for broader access to high-quality training tools without compromising educational value.

### Participants

The participants in this study were mainly medical students from the National Defense Medical University. The course is open to all students, not exclusively to those in medical departments, allowing for voluntary participation from various fields. All participants filled out an informed consent form before engaging in the lesson. The participants included 22 males and 8 females, with 24 from the Department of Medicine, 3 from the Department of Traditional Chinese Medicine, and 3 from the Department of Pharmacy. The group includes 19 first-year medical clerks (Clerk I), 10 s-year medical clerks (Clerk II), and one lecturer. The participants completed the Learning Satisfaction Questionnaire and the Confidence Perception Questionnaire. The detailed background of the participants is shown in Table 1.

**Table 1:** The characteristics of the participants

Department	Grade	Gender	Number of People
**Department of Medicine**	Clerk I	Male	15
		Female	4
	Clerk II	Male	4
		Female	1
**Department of Traditional Chinese Medicine**	Clerk II	Male	2
		Female	1
**Department of Pharmacy**	Clerk II	Male	1
		Female	1
	Lecturer	Female	1
Total			30

The researchers then recruited 13 interviewees from the participants above and the teaching assistants for interviews. Almost all of the interviewees were from the medical department, with only one student from the Department of Traditional Chinese Medicine. There were 12 males and 2 females, including 9 clerk I and 4 Clerk II, and 1 teaching assistant who was a resident physician. The detailed background of the interviewees is shown in Table 2.

**Table 2:** The background of the interviewees

Department	Grade	Gender	Number of People
Department of Medicine	Clerk I	Male	7
		Female	2
	Clerk II	Male	3
	Teaching Assistant	Male	1
Department of Traditional Chinese Medicine	Clerk II	Male	1
Total			14

### Lesson structure

The lesson began with the instructor explaining the skull’s structure and the brain tissues visible through the burr hole technique. Three training sessions were conducted with 7, 8, and 15 students respectively, each lasting 1.5–2 h, with more time for larger groups to ensure full participation. The lessons occurred outside an actual operating room, so no preparatory clinical work was discussed, and the instruments used were not sterilized, as there were no real patients involved.

#### Step 1. Burr hole

Before the burr hole procedure, the instructor had students mark the operation area and identify drilling sites. They explained the purpose, process, indications, and precautions of craniectomy, then allowed students to perform it. “Surgical Burr” refers to drilling, with the high-speed machine grinding holes into the skull. The instructor highlighted techniques to avoid meningeal bleeding in real surgeries. Each lesson featured PLB simulators, shared by 7–8 students, who took turns drilling, with each student spending about 5 min on the task.

#### Step 2. Decompressive craniectomy

**Fig. 2:**
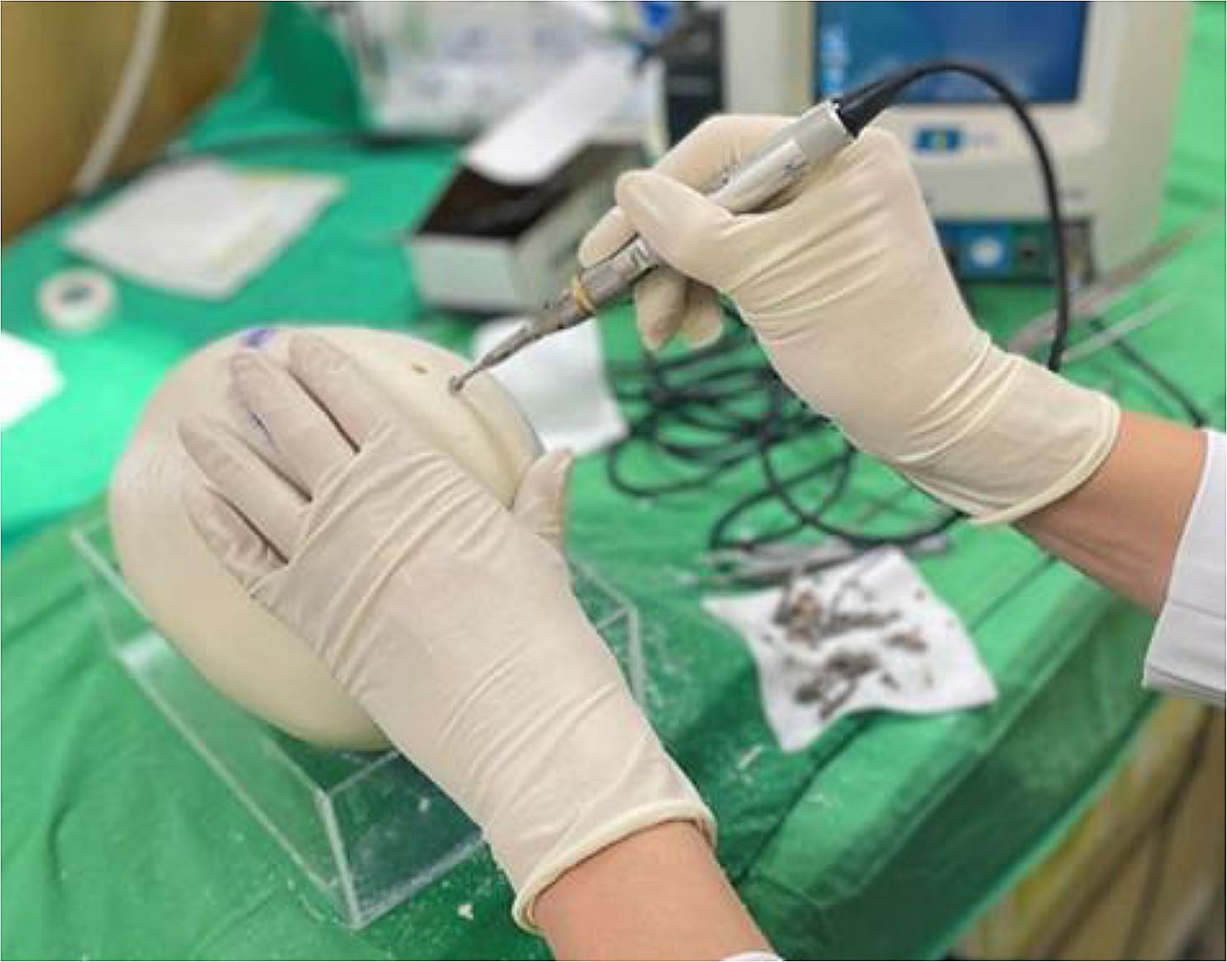
The surgical burr used in the innovative lesson on glioblastoma removal and decompressive craniectomy

The instructor outlined the craniectomy procedure before drilling into the PLB skull with a specialized drill. The instructor then connected the holes using a power saw to remove a skull piece. After observing these demonstrations, students started their hands-on practice. This lesson offered a more authentic clinical surgery experience compared to the gross anatomy course; the anatomy course used a saw resembling a pizza cutter, whereas this lesson employed a surgical burr resembling a pen with a front drill, mirroring tools used in real operating rooms (Fig. 2).

#### Step 3. Glioblastoma removal

**Fig. 3:**
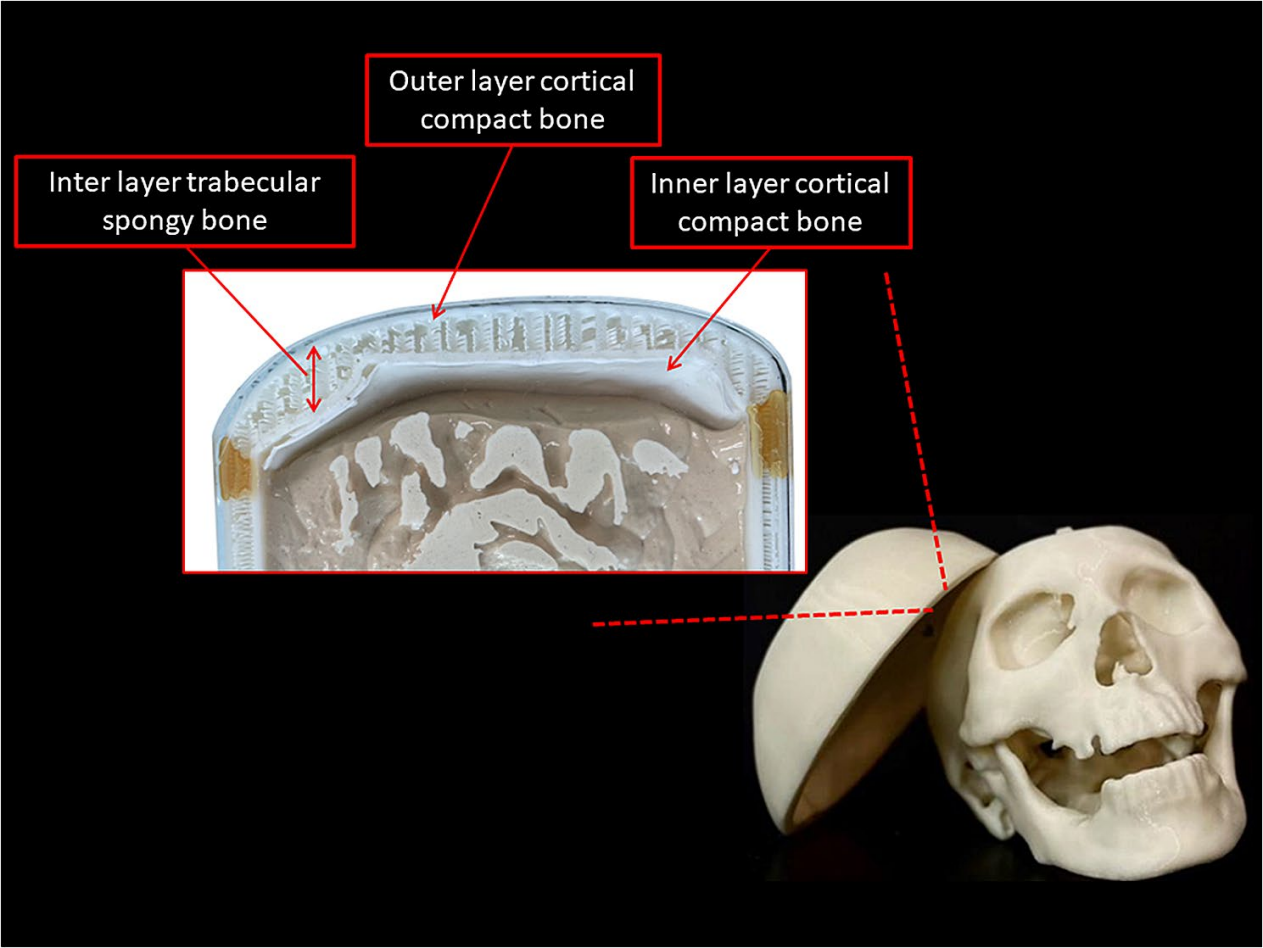
The students used a 3D-printed simulator for practice (only the skull)

The instructor used a 3D-printed simulator to demonstrate the process of glioblastoma removal and explain the typical locations of glioblastoma. It is worth noting that the 3D-printed simulator used by the instructor includes four parts, brain stem, brain lobe, and glioblastoma. During the drilling process, students utilized a 3D-printed simulator that had already been modified to remove the brainstem, brain lobes, glioblastoma, and other components, leaving only the skull portion for practice (Fig. 3). For the glioblastoma removal practice, they switched to a pre-prepared brain model, which included the relevant anatomical features for them to practice the removal process.

### Qualitative interview

Considering that the data collected by structured questionnaires cannot measure more sensitive and delicate psychological changes, qualitative interviews were conducted to compensate for this. Accordingly, this study adopted a semi-structured interview to deeply understand the uniqueness and complexity of the cases. The interview content was flexible and focused on the research topic. The interviewees could speak freely and naturally provide detailed opinions. The researcher first prepared a first draft of the interview outline according to the research purpose (Supplementary Information 1). To determine the appropriateness of the interview content, the researchers invited a neurosurgery specialist, a mechanical engineering professor specializing in 3D printing manufacturing, and a science education expert to review the interview outline. Students who completed the course more quickly were interviewed, whereas those who finished more slowly were not interviewed due to time constraints. After consent, interviews lasting 0.5–1 h were conducted, recorded, and transcribed. The method applied involved systematically categorizing interview content into themes for evaluation, aided by a peer with qualitative research experience. Similar concepts were organized into sub-categories, which were then consolidated into main categories. When interviewee responses spanned multiple themes, they were accordingly classified into several categories simultaneously. Interviews built upon each other to form conclusions. Transcripts were translated from Chinese to English by a fluent researcher and then back-translated to ensure accuracy, adjusting any discrepancies to validate the translation. Since the students took the gross anatomy course 2–4 years before the current lesson, the interview began by asking them about the content of the course, especially the units of the brain and skull. Students were then asked to evaluate the innovative course in terms of its difficulty, practicality, learning effectiveness, and learning environment.

### Instrument

#### Learning satisfaction questionnaire

The purpose of the learning satisfaction questionnaire was to determine if the students were satisfied with the training. The questionnaire’s content was taken from a course evaluation survey by Bohl [12]. These questions were answered using a 5-point Likert scale (very helpful, helpful, no opinion, only a little help, no help at all). The questionnaire was divided into 5 dimensions: lesson arrangement and design, instructor, teaching assistant, learning environment, and lesson contents. The detailed questionnaire content is shown in Supplementary Information 2.

### Confidence perception survey

Because the training was designed to improve surgical skills and reduce anxiety before surgery, a confidence perception questionnaire was designed. Trainees completed the survey before and after the training to determine if their confidence had improved. The questionnaire’s content was based on the study of Acosta et al. [13] and was modified based on the purpose of the training. The questionnaire included 5 items: (1) Preoperative preparation; (2) Check the equipment and instruments required for the operation; (3) Understanding and determining the scope of surgery; (4) Surgical disinfection and sterilization; (5) Making the burr hole. Questions were answered using a 5-point Likert scale. The survey question content is shown in Supplementary Information 2.

### Statistical analysis

In this study, a study of descriptive statistics of students’ learning satisfaction and confidence perception was conducted, reporting the mean and standard deviation (SD) of each questionnaire item. The questionnaire utilized several levels of satisfaction, ranging from “extremely helpful” to “without any help”. The proportion of students at each satisfaction level to the total student population was calculated and expressed as a percentage. Regarding students’ perception of confidence in surgical skills before and after the innovation lesson, a paired sample t-test was conducted to calculate the *t* and *p* values with the effect size, Cohen’s d also reported. All statistical tests were two-tailed and a p-value of less than 0.05 was considered statistically significant.

## Results

### Student satisfaction with the innovative lesson on glioblastoma removal and decompressive craniectomy according to the questionnaire

**Fig. 4:**
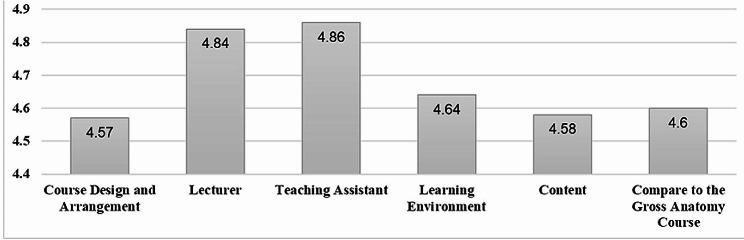
Students’ satisfaction with learning in different aspects

Based on the learning satisfaction questionnaire results, the mean satisfaction was 4.57 ± 0.45; the mean satisfaction with the instructors was 4.84 ± 0.35; the mean satisfaction with the teaching assistant was 4.86 ± 0.33; the mean satisfaction with the learning environment was 4.64 ± 0.54; the mean satisfaction with the teaching content was 4.58 ± 0.49. Students rated this course as helpful, with a mean of 4.6 ± 0.51. Overall, the mean student satisfaction score for the innovative training was 4.71 ± 0.34. Different aspects of student satisfaction are shown in Fig. 4.

**Fig. 5:**
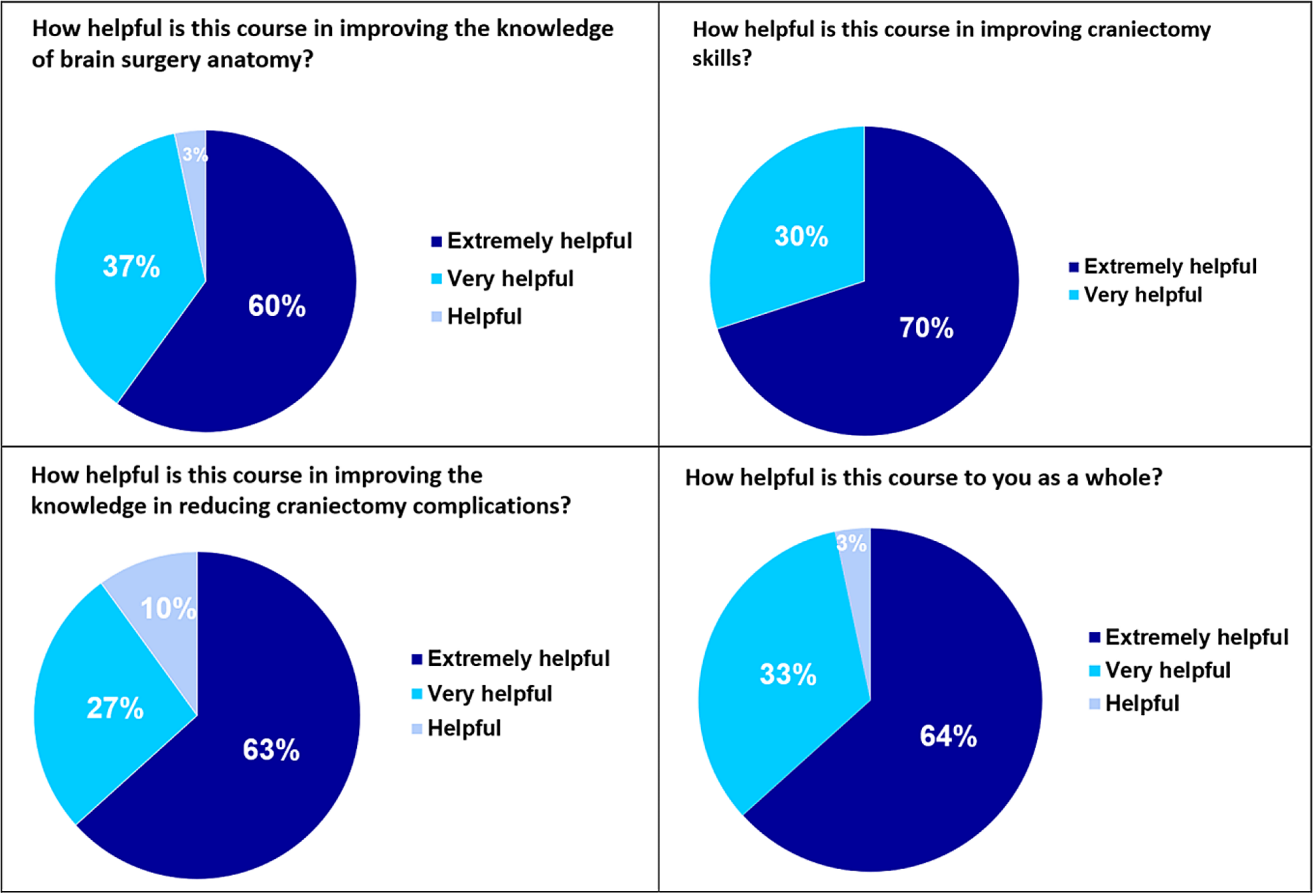
Students’ feedback on the benefits of the innovative lessons

On the basis of the knowledge gained form the gross anatomy class’s brain and skull units, 60% of the students thought this lesson was extremely helpful in improving their knowledge of neuroanatomy in brain surgery, 37% of the students thought it was very helpful, and 3% of the students thought it was helpful. In addition, 70% of the students thought this lesson was extremely helpful in improving the craniectomy skills, and the other 30% thought it was very helpful. For improving the knowledge in reducing craniectomy complications, 63% of the students thought this lesson was extremely helpful, 27% thought it was very helpful, and 10% thought it was helpful. 64% of students found this lesson as a whole extremely helpful, 33% found it very helpful, and 3% found it helpful (Fig. 5.)

### The improvement of students’ perception of confidence in surgical skills

The mean score of perception of confidence in surgical skills improved from 2.15 ± 1.02 before the training to 3.59 ± 0.93 after the training (t = 9.38, *p* < 0.001, effect size = 1.48). The mean score of confidence in preoperative preparation improved from 2.30 ± 1.15 before the training to 3.53 ± 1.04 after the training (t = 6.50, *p* < 0.001, effect size = 1.12). The perception of confidence in checking the equipment and instruments required for surgery improved from 2.03 ± 1.10 before the training to 3.47 ± 1.04 after the training (t = 8.75, *p* < 0.001, effect size = 1.35). Student perception of confidence in understanding and determining the scope of surgery improved from 2.33 ± 1.16 before the training to 3.57 ± 1.04 after the training (t = 8.75, *p* < 0.001, effect size = 1.13). Student perception of confidence in surgical disinfection and sterilization improved from 2.37 ± 1.22 before the training to 3.67 ± 0.99 after the training (t = 7.21, *p* < 0.001, effect size = 1.17). Student perception of confidence in performing a craniectomy increased from 1.73 ± 1.05 before the training to 3.70 ± 0.99 after the training (t = 11.61, *p* < 0.001, effect size = 1.93) (Table 3).

**Table 3:** Analysis of students’ confidence perception scores

	Pre-test	Post-test	t	*p*	Cohen’s d
	Mean *±* SD	Mean *±* SD			
**Preoperative preparation**	2.30 *±* 1.15	3.53 *±* 1.04	6.50	< 0.001	1.12
**Check the equipment and instruments required for surgery**	2.03 *±* 1.10	3.47 *±* 1.04	8.75	< 0.001	1.35
**Understand and determine the scope of surgery**	2.33 *±* 1.16	3.57 *±* 1.04	6.30	< 0.001	1.13
**Surgical disinfection and Sterilization**	2.37 *±* 1.22	3.67 *±* 0.99	7.21	< 0.001	1.17
**Perform a craniotomy**	1.73 *±* 1.05	3.70 *±* 0.99	11.61	< 0.001	1.93
**Average**	2.15 *±* 1.02	3.59 *±* 0.93	9.38	< 0.001	1.48

### Summary results of the qualitative interview

Interview results indicated that: (1) This innovative lesson is different from the gross anatomy class on the skull in terms of the knowledge regarding brain surgery anatomy. The gross anatomy courses guided students to understand the relative anatomical positions of different brain tissues, which were more realistic than the abstract anatomic atlas. However, when students dissected the skull in the gross anatomy course, they did not particularly notice that it has three different layers. In contrast, this innovation lesson helped students understand the different layers of the skull, the relative positions of the meninges and the skull, and where the blood clots may occur during brain hemorrhages. (2) Formalin negatively affects student satisfaction in cadaver-based learning due to its strong odor and interference with tissue identification. In prior gross anatomy class, students needed to clean the sink and restore the environment, reducing their satisfaction with the learning environment. Notably, the PLB simulator does not require formalin and is not heavy like a cadaver. Hence, the learning environment is much more comfortable. The qualitative interview results are detailed in Supplementary Information 3.

## Discussion

Ryan et al. used 3D printing and silicone casting to develop a patient-derived medical simulator for aneurysm clipping training [14]. Through a team of neurosurgeons, simulation engineers, and special effects experts, Weinstock et al. developed a full-scale simulation model of the head of a 14-year-old adolescent hydrocephalus patient for neurosurgery training of new residents [15]. The above studies demonstrate the effectiveness of 3D-printed and cast-fabricated simulators for medical research and training. In this study, we optimized a PLB simulator previously developed by 3D printing and casting technology by our research team [16, 17]. The optimized PLB simulator, made using specific methods and material ratios, aims to offer medical students a tangible training tool to improve surgical education.

Students gave the innovative lesson on glioblastoma removal and decompressive craniectomy high marks, with an average satisfaction score of 4.71. Scores in all evaluated areas, including lesson organization, teaching staff, learning environment, and content, were above 4.5, indicating high levels of student satisfaction. Moreover, over 60% of students rated the lesson as “extremely helpful” for improving brain surgical anatomy knowledge, craniotomy skills, and knowledge in reducing craniotomy complications, demonstrating its effectiveness in their learning.

Before the innovative training, students were not confident about their skills in the critical steps associated with the surgery. The study results are consistent with findings from previous studies where general surgery residents expressed concerns about surgery-related skills due to a lack of surgery-related training [18, 19]. Post-training, students reported enhanced confidence across five key areas: preoperative preparation, equipment and instrument verification, understanding the surgery’s extent, surgical disinfection and sterilization, and executing a burr hole. Notably, confidence in performing a burr hole rose from a mean of 1.73 to 3.70, with an effect size of 1.93, demonstrating the training’s positive impact on surgical skill confidence. Looking ahead, incorporating cadaveric brain tissue into the PLB simulator is considered to further align the anatomical experience with real surgical conditions.

Burr-hole craniectomy is a common neurosurgical approach for cerebral hemorrhage and hematoma [20–23]. This innovative decompressive craniectomy and glioblastoma removal practice via 3D-printed models is helpful to medical students in gaining an initial understanding and provoking their interest in these neurosurgical techniques. Particularly, the realism of the PLB simulator increases the usefulness of the innovative training. Moreover, due to the high cost and limited availability of cadavers, not every student gets hands-on experience with brain removal in gross anatomy courses. In contrast, PLB simulators are significantly cheaper, allowing for the provision of more units and increased practice opportunities for students [24, 25] Further, due to the formalin preservation, the cadaver’s brain shrank, deviating from the real appearance in the clinical practices. In contrast, the PLB simulator offered a more lifelike experience in a way.

Collectively, the students recognize the value of 3D-printed simulators in medical education. The positive feedback from students underscores the critical role of this novel approach in surgical training. Future efforts should focus on creating affordable simulators that incorporate detailed brain structures, including the meninges and brain soft tissues, nerves, or brain microvessels, allowing trainees to extensively practice the procedures in a manner that closely mimics real-life conditions. Additionally, the aim is to standardize and commercialize these teaching aids, making them widely available. By systematically integrating these tools into the medical curriculum, we can enhance educational outcomes.

### Strengths and limitations

The key advantage of this study lies in the development of an innovative educational tool that combines accessibility with cost-effectiveness, establishing a sustainable model for learning that leverages reusable resources. Furthermore, the study has gathered evidence confirming the tool’s efficacy it delivers in educational and training contexts. However, this study has several limitations. Firstly, there was a 2 to 4-year gap from the students’ gross anatomy class to this innovative lesson, potentially hindering the continuity and immediacy of its efficacy. This temporal gap might also bias their assessment regarding the benefits derived from this course. Secondly, only students who completed the course quickly were interviewed, potentially introducing bias if these students were either less interested or better prepared than their peers. Thirdly, while qualitative methods provide depth to the learning experience, they are subjective and might not capture the full spectrum of students’ learning outcomes. Moreover, although these students found the practices helpful, they were not able to compare it with real-world scenarios as they had limited experience with the actual surgeries. Finally, the study’s design and statistical power are constrained by the sample size, which may limit the generalizability of the findings. These limitations underscore the need for a cautious interpretation of the results.

## Conclusion

We developed a cutting-edge course focused on craniotomy and decompressive craniectomy techniques, utilizing 3D-printed skull models for simulation. Feedback indicated that students greatly valued the course, noting a substantial boost in their confidence regarding these procedures. To sum up, this pioneering course effectively enhanced the craniotomy-related knowledge and practical skills of the students.

### Electronic supplementary material

Below is the link to the electronic supplementary material.

Supplementary Material 1

Supplementary Material 2

## Data Availability

The datasets used and/or analyzed during the current study are available from the corresponding author on request.

## Abbreviations

PLB: Physical Lifelike Brain
TMZ: Temozolomide
MRI: Magnetic resonance Imaging
Computerized tomography: CT
ABS: Acrylonitrile butadiene styrene
