# Supplementary material for: Improving medical students recognizing surgery of glioblastoma removal/decompressive craniectomy via physical lifelike brain simulator training
Source: BMC Med Educ. 2024 Jun 6;24:632. doi: 10.1186/s12909-024-05621-w (PMC11155129; doi:10.1186/s12909-024-05621-w)
Supplement: Supplementary file 2 — Supplementary Material 2 [file 12909_2024_5621_MOESM2_ESM.docx]

# SUPPLEMENTARY INFORMATION 1

## Interview outline

1. **What have you been taught in past gross anatomy classes? Make a statement about the brain (or skull).**
   1. **Have you done any of the following tasks in your previous gross anatomy course?**
      1. Preoperative preparation and inspection equipment.
      2. Determine the scope of surgery.
      3. Glioblastoma removal.
      4. Burr hole craniotomy.
      5. Decompressive craniectomy.
   2. **For the brain (or skull) unit, please answer in order:**
      1. Difficulty for the brain (or skull) unit.
      2. Hours for brain (or skull) units.
      3. Utility for Brain (or Skull) Units
      4. What are the textbooks/slides used for the brain (or skull) unit?
   3. **For the brain (or skull) unit of the gross anatomy unit, the learning benefits of the course:**
      1. In your case, did the gross anatomy course help improve your knowledge of brain surgery anatomy? Why?
      2. In your case, did the gross anatomy course help improve your decompressive craniectomy technique? Why?
      3. In your case, did gross anatomy courses help improve the complications of craniotomy

in patients? Why?

- - 1. In your case, did the gross anatomy course help you in other ways?

1. **What did you learn in the "Innovative Lesson on Decompressive Craniectomy"?**
   1. **Did you do the following tasks in this lesson?**
      1. Preoperative preparation and inspection of instruments.
      2. Determine the scope of surgery.
      3. Glioblastoma removal
      4. Burr hole craniotomy
      5. Decompressive craniectomy
   2. **For this lesson, please answer in order:**
      1. The difficulty level of this lesson.
      2. Hours of this lesson.
      3. The practicality of this lesson.
      4. What are the contents of this lesson textbook/slideshow?
   3. **Learning benefits of this lesson:**
      1. In your case, did this lesson help improve your knowledge of brain surgery anatomy? Why?
      2. In your case, did this lesson help you improve your decompressive craniectomy technique? Why?
      3. In your case, did this lesson help improve the complications of craniotomy in patients?

Why?

- - 1. As far as you are concerned, has this lesson helped you in other ways?

1. **Comparing Gross Anatomy with this lesson**
   1. **Please compare this lesson with the Gross Anatomy course and describe the difference between the two classes in detail. (What are the advantages and disadvantages)**
   2. **Comparison of student learning benefits**
      1. Please compare this lesson with the Gross Anatomy course. As far as you are concerned, which class was most helpful in improving your knowledge of brain surgery anatomy? Why?
      2. Please compare this lesson with the Gross Anatomy course. In your case, which class was most helpful in improving your decompressive craniectomy technique? Why?
      3. Please compare this lesson with the Gross Anatomy course. As far as you are concerned, which class was most helpful in improving the complications of craniotomy in patients? Why?
   3. **Please compare this lesson with the Gross Anatomy course. As far as you are concerned, what are the differences in the learning environment? (Example: Ventilation, smell, brightness, experimental equipment...etc.)**

# SUPPLEMENTARY INFORMATION 2

## Learning Satisfaction Questionnaire

- - - 5 = Extremely helpful
    - 4 = Very helpful
    - 3 = Helpful

| - 2 = Little help - 1 = Without any help |  | | | |
| --- | --- | --- | --- | --- |
|  | Scale |  |  |  |
| **Investigation** | 5 4 | 3 | 2 | 1 |
| **1. Curriculum Arrangement and Design** |  |  |  |  |
| (1) Lesson arrangement |  |  |  |  |
| (2) Difficulty of the lesson |  |  |  |  |
| (3) Teaching hours |  |  |  |  |
| (4) Practicality of the lesson |  |  |  |  |
| (5) Lessonmaterial/slideshow content |  |  |  |  |
| (6) Overall satisfaction with lesson arrangement and design |  |  |  |  |
| **2. Instructor** |  |  |  |  |
| (1) Instructor's expertise |  |  |  |  |
| (2) Instructor's ability to answer questions |  |  |  |  |
| (3) Interaction with the instructor |  |  |  |  |
| (4) Instructor's expressive ability |  |  |  |  |
| (5) Teaching attitude of the instructor |  |  |  |  |
| (6) Instructor's attendance |  |  |  |  |
| (7) Overall satisfaction with the instructor |  |  |  |  |
| **3. Teaching Assistant (TA)** |  |  |  |  |
| (1) Professional knowledge of TA |  |  |  |  |
| (2) The ability of the TA to answer questions |  |  |  |  |
| (3) Interactive with TA |  |  |  |  |
| (4) Presentation skills of TA |  |  |  |  |
| (5) Teaching attitude of TA |  |  |  |  |
| (6) Attendance status of TA |  |  |  |  |
| (7) Overall satisfaction with TA |  |  |  |  |
| **4. Learning environment** |  |  |  |  |
| (1) Cleanliness of the learning environment |  |  |  |  |
| (2) Teaching equipment (tables and chairs, laboratory equipment...etc.) |  |  |  |  |
| (3) The light, brightness, and ventilation of the teaching environment |  |  |  |  |
|  | Scale |  |  |  |
| **Investigation** | 5 4 | 3 | 2 | 1 |
| **5. Lesson Contents** |  |  |  |  |
| (1) As far as you are concerned, how helpful has this lesson improved |  |  |  |  |

your knowledge of brain surgery anatomy?

1. As far as you are concerned, how helpful has this lesson been in

improving the technique of decompressive craniectomy?

1. As far as you are concerned, how helpful has this lesson been in improving the technique of complications of craniotomy?
2. Overall, how helpful has this lesson been to you?
3. Compared to the gross anatomy course, how helpful has this lesson been in improving your knowledge of brain surgery anatomy as far as you are concerned?
4. Compared to the Gross Anatomy course, how helpful has this lesson been in improving your decompressive craniectomy technique as far as you are concerned?
5. Compared to the gross anatomy course, in your case, how helpful has this lesson been in improving the complications of craniotomy in patients?
6. Compared to the Gross Anatomy course, how helpful has this lesson been to you as a whole?

***1.2 Confidence Perception Survey***

The following questions are about your confidence in the skills you have acquired in the classroom. The results of this test are for research purposes only and are not included in your academic performance. Please feel free to answer.

Please answer according to your actual feeling and situation after reading carefully. There is no absolute right or wrong answer. Please rate your confidence level in the following tasks on a scale of 1-5 below. All questions are multiple-choice questions. Please tick "☑" in the most appropriate box.

**Skills related to surgery and key steps**

- 5 = I can complete the task independently and proficiently.
- 4 = I am confident in doing the task but still need some supervision.
- 3 = I have some experience with the task but still need some aid.
- 2 = I have little experience with the task and lack confidence.
- 1 = I do not have experience with the task and cannot complete the task.

Scale

5 4 3 2 1

1. Preoperative preparation
2. Check the equipment and instruments required for surgery
3. Understand and determine the scope of surgery
4. Surgical sterilization and a sterile drape
5. Craniotomy

# SUPPLEMENTARY INFORMATION 3

### Qualitative Research: Summary of Interview Results

| **Theme** | **Summary of Findings** | **Representative Quotes** |
| --- | --- | --- |
| Difficulty of the Course | In this innovation lesson, careful craniotomy area selection and craniotomy skills are crucial to avoid injuring brain tissue. | “In the gross anatomy course, the skull has been removed entirely, and the brain tissue can be seen easily. In contrast, the skull is still there in clinical operations, and the brain tissue can only be seen from a specific hole and angle." - Interviewee 4 |
| Usefulness of the Course | The innovative lesson was found more useful for clinical practice and emergency scenarios. | "Not only neurosurgery specialists but also emergency residents may find it useful." - Interviewee 1 |
| Learning Environment | The innovative lesson offered a more comfortable learning environment without the formalin odor and heavy cadavers. | "The 3D-printed skull simulator of the innovative lesson was clean; it had no smell. The brain tissue was visible clearly." - Interviewee 12 |
| Improvement in Anatomical Knowledge | Both courses were helpful, but the innovative lesson provided a better understanding of the layers of the skull. | "The simulator showed all three layers of the skull clearly, which helped us understand the anatomy better." - Interviewee 7 |
| Enhancement of Surgical Skills | The innovative lesson significantly improved students' practical skills, especially in burr hole techniques. | " The lesson gave us the experience that when we encounter a patient in the future, we can simulate the operation process in our mind first to make the operation more precise. " - Interviewee 9 |
| Avoidance of Complications in Craniotomy | Practicing with the simulator helped students understand how to avoid complications during surgery. | "Practicing on the simulator helped avoid potential complications." - Interviewee 1 |
| Overall Educational and Career Benefits | The innovative lesson enhanced interest and knowledge in neurosurgery, providing a realistic simulation experience. | "I knew the burr hole technique is a primary or essential neurosurgery technique. I heard that 50% of neurosurgery procedures require this technique." - Interviewee 11 |

### Qualitative Research: Details of Interview Results

- 1. ***The difference between the difficulty of the gross anatomy course and the innovative lesson***

According to the interview results, 9 interviewees thought the gross anatomy course was challenging. The reason is that the brain's structure is complex, and the functions of each part are different. There are anatomic atlases in the textbooks, and the different structures are marked with different colors. However, the brain structures of the cadaver are not significantly different in color and can only be identified by the texture. Therefore, it is relatively difficult to identify the anatomical structure of the cadaver's brain section.

*Interviewee 1: Some structures are difficult to identify. In the anatomy book, the structures are specially marked in color. However, the color will be almost the same when seen on the cadaver, and it is not easy to recognize the structure. You need to distinguish the structure by texture or position.*

*Interviewee 7: The skull was removed and contained meninges and brains. The brain part was then sliced horizontally and vertically and compared to the neural anatomic atlas. In the anatomic atlas, the structure was marked where it is in the brain and to which structure it belongs. (e.g., the hippocampus). I think comparing the anatomic atlas, it is harder to identify the anatomical position of the brain in the cadaver.*

In addition, four interviewees compared gross anatomy with actual clinical situations. In clinical operations, attention must be paid to the area and angle of the craniotomy to reduce the possibility of damaging the patient's brain. Therefore, only one area would be dug out in the brain. Skull was still there, and the brain tissue had to be viewed from a specific angle. In contrast, in the gross anatomy course, the skill is removed entirely without regard for possible damage. Therefore, the brain tissue can be easily seen with much less difficulty.

*Interviewee 4: In the gross anatomy course, the brains and nerves are taken out of the skull, so it is not difficult to recognize them. However, we only dig a hole in the skull in actual operations. Therefore, you can only see the brain tissue from specific angles. In other words, in the gross anatomy course, the skull has been removed entirely, and the brain tissue can be seen easily. In contrast, the skull is still there in clinical operations, and the brain tissue can only be seen from a specific hole and angle.*

Most interviewees agreed that the difficulty depends on their proficiency of using surgical burs for the innovation lesson on craniotomy with the removal of glioblastoma and decompressive craniectomy. Among them, 9 interviewees believed that the lesson emphasized the practice of using the surgical burs, so the difficulty level was moderate. Two other interviewees indicated that the teaching assistant's demonstration reduced the lesson's difficulty.

*Interviewee 8: at first, I had no idea how to operate the surgical burr. Fortunately, after watching the teaching assistant operate, I finally figured out how to do it. In other words, I initially felt quite abstract and challenging. But after watching the teaching assistant's demonstration, it became straightforward.*

### The difference between the usefulness of the gross anatomy course and the innovative lesson

According to the interview results, most interviewees said that the gross anatomy course has some practicality for their future national examination. However, 6 interviewees thought that the clinical usefulness of the gross anatomy course was not high. It is useful for the national examination because the national examination questions focus on anatomy-related knowledge. Seeing the 'actual' structure of the human body is more beneficial for anatomy than seeing the 'abstract' structure on the anatomic atlas.

*Interviewee 4: gross anatomy courses are helpful for national exams and for building basic concepts.*

*Interviewee 7: Compared with textbooks, anatomy still needs to be seen and touched, and it will be more impressive.*

6 interviewees believed that the reason for the low clinical practicability of the gross anatomy course was that the head seen by gross anatomy is directly incised; however, clinically, the part is identified through an endoscope or a surgical perspective. The two views are very different. As a result, students still do not know how to deal with practical problems clinically in the future after engaging in the course.

*Interviewee 6: It is better to see the surgery clinically to Identify the site through an endoscope or a surgical perspective. The brain was cut open in the gross anatomy course directly in front of us. The two views are quite different.*

In terms of the practicality of the innovative lesson, 6 interviewees believed that the lesson is only practical for neurosurgery students. However, 3 interviewees said this lesson is useful for neurosurgery students and may also be used in emergencies (e.g., cerebral hemorrhage and cerebral edema).

*Interviewee 1: Not only neurosurgery specialists but residents in the emergency department may also encounter patients with cerebral hemorrhage. After graduation, we must go to various departments before determining a department to stay in. In that case, we will also need to go to the emergency room. In the emergency department, you may encounter decompressive craniectomy. In other words, no matter what department you finally determine, you have the chance to meet such a situation.*

*Interviewee 9: Some students are going to hospitals in the countryside. If the patient happens to have cerebral edema, urgent decompressive craniectomy is required. Before sending him to the big hospital, we first performed a decompressive craniectomy to reduce the patient's brain pressure, which could help stabilize the patient's condition before handing him*

*over to the neurosurgeon at the city hospital for further surgery.*

The other 2 interviewees said that the simulator in the lesson is very realistic, allowing students to experience the actual situation in the operating room. Therefore, the practicability of the lesson is very high.

*Interviewee 12: Because of the simulator, we can practice the burr hole technique. The skulls are realistic, and every layer is visible. Practicing the burr hole technique before performing this operation on the patient will also be much better for the patient. At least with experience, you will not be so scared.*

*Interviewee 13: Because there are instruments for operating burr holes, we know how much force we must use to drill and how to position it to make it more stable. Therefore, I think the lesson is very useful.*

### The difference between the learning environment of the gross anatomy course and the innovative lesson

Comparing the two learning environments, most interviewees said that the gross anatomy course had a strong smell of formalin, but Burr hole's 3D printed skull simulator did not have the smell of formalin. Interviewees also said that besides the strong smell of formalin, there were many exudates, making it difficult to see the tissue and accidentally cut it.

*Interviewee 12: There was a strong odor of formalin in the gross anatomy course. The exudate of formalin made the tissue less visible, causing it to be inadvertently cut off. The*

*3D-printed skull simulator of the innovative lesson was clean; it had no smell. The brain tissue was visible clearly.*

*Interviewee 7: Most cadavers were soaked in formalin, and the smell of formalin in the whole laboratory was strong. The formalin made me feel a little tingling, runny nose, and tears, and the smell of formalin stuck to my body for a whole day. The gross anatomy course had a place similar to the sink where the cadaver lies. So there will be some formalin oozing out as we work.*

Regarding the teaching aids, 2 interviewees said that the cadaver used in the gross anatomy course was heavy, and the handling process was not easy. Also, cleaning the table and restoring the environment took some time after completing the gross anatomy course.

*Interviewee 3: The innovative lesson was more convenient to handle. In the gross anatomy course, the cadaver must be moved, the head was hefty, and sometimes the angle of the cadaver needed to be adjusted, which was more cumbersome to carry.*

*Interviewee 4: After the gross anatomy class, you had to wash the table, restore the environment and clean up the rotten meat, but not in the innovative lesson.*

The PLB simulator of the innovative lesson on glioblastoma removal and decompressive craniectomy had some crumbs generated by grinding the skull.

*Interviewee 2: One disadvantage of the innovative lesson is that the bone scraps were sprayed all over the place, making the learning environment less comfortable. Since the bone*

*scraps are so light, they were scattered around our hair.*

Regarding the number of teaching aids, 2 interviewees indicated that there were more than 100 students in the gross anatomy course and only a few cadavers. However, the innovation course on glioblastoma removal and craniotomy offered more physical simulators.

*Interviewee 9: The space for the gross anatomy course was a little crowded and relatively small, as there were about 10 people in a group. We sometimes needed to share the instruments, such as scissors, blades, and skull knocking. We could not use those instruments until the other group members were done using them.*

*Interviewee 13: The advantage of the innovative lesson should be the number part. In the innovative lesson, a group has just about 6 students, less than half the size of the gross anatomy course. There are also more instruments provided so that I can practice more times. However, we can only practice parts of the cadavers in the gross anatomy course.*

*Interviewee 11: The Department of Traditional Chinese Medicine had no opportunities to practice. We could only see the cut tissue made by medical students.*

### The difference between the gross anatomy course and the innovative lesson in improving the anatomy knowledge of brain surgery

In terms of helping to improve the knowledge of brain surgery anatomy, most of the interviewees said that the gross anatomy course was helpful to students' understanding of brain surgery anatomy. 5 interviewees said the cadaver is a three-dimensional and real anatomical structure instead of the textbook's flat and abstract anatomic atlas. Thus, it helped students understand surgical anatomy more.

*Interviewee 2: Anatomy is very three-dimensional stuff. If you actually see or touch it, you will have a better concept of three-dimensional space and understand it better than just looking at an anatomic atlas. For example, the texture of the brain tissue, you can only feel it when you touch it. When looking at the anatomic atlas, you must use your imagination. The anatomic atlas is very abstract; however, the cadaver is what you actually see!*

Another 6 interviewees said that the gross anatomy course gave students a basic idea of the relative positions of different brain tissues.

*Interviewee 3: the gross anatomy course helped us better understand what the anatomy of the brain, such as gray and white matter and cerebellum. Moreover, we would gain a more conceptual understanding of cranial nerves and their relative positions.*

Most interviewees indicated that it differed from the gross anatomy course in helping understand the anatomy of brain surgery for the innovation lesson on glioblastoma removal and decompressive craniectomy. The PLB simulator of the innovative lesson was very realistic, allowing students to know the 3 layers of the skull, the relative position of the meninges and the skull, as well as the possible locations of cerebral hemorrhage and glioblastoma. However, gross anatomy focuses on the anatomy of the brain tissue.

*Interviewee 1: The gross anatomy course focused more on the brain tissue. However, the*

*innovative lesson helps us to see not only the state of the brain at the time of surgery but also learn more about the relative position of the bones.*

*Interviewee 7: Because the simulator was made very delicate, all three layers of the skull were also visible. The outermost layer was relatively dense bone (cortical bone), the middle layer was like a sponge, and the innermost layer was also relatively dense cortical. As far as anatomy is concerned, I think it is quite helpful. The gross anatomy course aims to help us identify the tissues in the brain. I only focused on knocking out the skull without looking at the layers of the skull, not to say figuring out the difference between the three layers.*

Compared with the gross anatomy course and the innovative lesson, 4 interviewees said the gross anatomy course was helpful for understanding the anatomical knowledge of 'normal' physiological structures. In contrast, the innovative lesson was more helpful for understanding the anatomy of clinical 'pathological' structures.

*Interviewee 7: gross anatomy looks at the normal physiological structure of cadavers, while what is seen clinically is a pathological structure, which is different from what is seen in gross anatomy.*

The other three interviewees indicated that the gross anatomy course helps students have a more comprehensive understanding of brain anatomical structures, while the innovative lesson focused on introducing selected anatomical areas.

*Interviewee 9: In the gross anatomy course, we started with the skull and then looked at the structure of the 12 pairs of cranial nerves; however, the innovative lesson is more one-sided, looking only at the structure of the skull.*

### The difference between the gross anatomy course and the innovative lesson in improving the skills of decompressive craniectomy

In terms of the help of decompressive craniectomy, most interviewees said that the gross anatomy course is different from clinical surgery, so it is of little help to improve students' skills in decompressive craniectomy.

*Interviewee 7: The cadavers in the gross anatomy course had been soaked in formalin, and they all shrank slightly. The brain was much smaller than the skull instead of a swollen state. However, the purpose of a decompressive craniectomy is to remove the pressure from the swollen brain. The head seen in gross anatomy was not under stress, which is different from the patient's brain. So I do not think the gross anatomy helps much in improving students' skills in decompressive craniectomy.*

Only 3 interviewees believed that the gross anatomy course helped improve students' skills in decompressive craniectomy because it could help students understand the relative positions of brain anatomy.

*Interviewee 10: You must understand the brain's anatomy before deciding the operation's extent and where to cut in.*

However, all interviewees agreed that the innovative lesson helped improve students' skills

in decompressive craniectomy. Among them, 8 interviewees said that the lesson gave them practical experience in burr hole technique.

*Interviewee 9: The lesson gave us the experience that when we encounter a patient in the future, we can simulate the operation process in our mind first to make the operation more precise.*

In addition, four students said that the burr hole technique is the first step of decompressive craniectomy, so practicing the burr hole technique helped students improve their skills in decompressive craniectomy.

*Interviewee 7: Craniotomy first requires a burr hole. Make a hole first, then cut it open with scissors or something. The burr hole technique is always used in decompressive craniectomy, so I think the lesson is helpful for students in improving their skills in decompressive craniectomy.*

Compared with the gross anatomy course, most students indicated that the innovative lesson was more helpful for students in improving their skills in decompressive craniectomy. Among them, 4 interviewees said the innovative lesson gave students actual operation experience, but the gross anatomy course did not.

*Interviewee 2: The innovative lesson is more helpful because it allows us to practice in burr hole technique, which the gross anatomy course did not.*

Five interviewees indicated that gross anatomy courses did not teach craniotomy-related content.

*Interviewee 9: We did not know there was decompressive craniectomy when we performed the gross anatomy. The teacher only asked us to cut the skull; the purpose was to let us recognize the 12 pairs of cranial nerves and the structure of the brain tissue. We were only in the second grade then, so we were mainly teaching essential medical content, and the clinical content only started in the third grade. So I think the innovative lesson is more like clinical content.*

One interviewee said that the craniotomy method of the gross anatomy course was different from the actual clinical practice.

*Interviewee 5: innovative lessons are more helpful than the gross anatomy course. The way of craniotomy in the gross anatomy course is different from that in clinical surgery.*

- 1. ***The difference between the gross anatomy course and the innovative lesson in improving the* craniotomy with the removal of glioblastoma *complications***

In terms of helping patients improve complications of craniotomy with the removal of glioblastoma, almost all interviewees said that the gross anatomy course was not very helpful in reducing patients' complications of craniotomy. Among them, 3 interviewees noted that the brain structure of the cadaver is normal physiological, while the brain structure of the craniotomy patient is pathological. Therefore, they are so different that the gross anatomy course is not helpful for clinical operations.

*Interviewee 9: postoperative complications are still related to the patient's condition.*

*However, the cadaver is not the patient, so the gross anatomy course is not so relevant for improving the patient's craniotomy complications.*

However, most interviewees agreed that the innovative lesson on craniotomy with removing glioblastoma and decompressive craniectomy helped improve patient complications from craniotomy. These interviewees indicated that the burr hole practice gave them a

hands-on opportunity to learn what to pay attention to when grinding the skull, thus avoiding possible complications.

*Interviewee 1: The innovative lesson taught us what to do when grinding the skull to avoid possible complications. Because if we do not know how to handle the force exactly, complications can occur. After the actual operation, you will learn more about when to reduce the force.*

*Interviewee 13: If you drill too deep, you will injure part of the dura. However, if you take a PLB simulator to practice the burr hole technique first, you will know the strength and depth, which will avoid harm to the patient. So I think the innovative lesson is pretty helpful.*

Compared with the gross anatomy course, 6 interviewees believed that the innovative lesson on glioblastoma removal and a craniotomy was more helpful in improving the complications of craniotomy. Another 3 interviewees said that the gross anatomy course and the innovative lesson helped differently. The gross anatomy courses taught the knowledge of anatomy; the innovative lesson is an exercise in surgical technique.

*Interviewee 7: The gross anatomy course was more like giving us an impression and basis of the brain anatomy so that we will not see these anatomical structures for the first time in clinical practice. However, this innovative lesson helped us know the clinical terminology, the burr hole technique, and when we need to use these procedures. Therefore, both of them are helpful for guiding us on how to improve patients' craniotomy complications.*

Finally, two students suggested that brain tissue could be added to the PLB simulator in the innovative lesson, which would be more helpful in guiding students on how to improve patients' craniotomy complications.

*Interviewee 7: I suggest that meninges can be added to the PLB simulator to make the drilling feels closer to reality.*

### The difference between the gross anatomy course and the innovative lesson in helping students with their studies or careers

In terms of helping students in their academic or future careers, most students said that the gross anatomy course could help them understand basic anatomy knowledge, which will be helpful for the national medical licensing examination.

*Interviewee 6: These lessons are somewhat helpful for anatomical knowledge. You can compare the anatomic atlas with the nerves, blood vessels, and muscles. Touching the actual brain tissue helps strengthen the impression of anatomical knowledge. It is the difference*

*between the abstract anatomic atlas and the real cadaver.*

In the innovative lesson, 8 interviewees believed that the lesson could help them understand craniotomy and the burr hole technique.

*Interviewee 7: Because the burr hole technique is clinically essential, actually using this instrument gave us a better idea of what it would feel like to use it.*

*Interviewee 10: Unlike just watching from the sidelines without a sense of participation, after engaging in the innovative lesson, we are able to know the actual operation feeling before entering the operating room in the future.*

1. interviewees said they increased their knowledge and interest in neurosurgery.

*Interviewee 11: I knew the burr hole technique is a primary or essential neurosurgery technique. I heard that 50% of neurosurgery procedures are required this technique.*

Two other interviewees said they found the importance of 3D printed simulators in medical education and were interested in the process of making 3D printed simulators.

*Interviewee 2: I am also interested in 3D printing fabrication. I am curious about what material the brain tissue is made of.*

*Interviewee 3: Besides knowing more about craniotomy, I figure out that 3D printing fabrication is essential in these simulation teachings! The teaching assistant also mentioned that the feel of the physics simulator is very similar to that of a real head.*
